# GCMS-based evaluation, characterization and in silico analysis of antimicrobial metabolites in the crude extract of cold-adapted *Alcaligenes pakistanensis* LTP10

**DOI:** 10.3934/microbiol.2026011

**Published:** 2026-05-06

**Authors:** Imran Rabbani, Muhammad Rafiq, Suliman Shah, Tariq Ahmad, Muhammad Irfan, Aamer Ali Shah, Turki M. Dawoud, Fariha Hasan

**Affiliations:** 1 Department of Pharmacy, Kohat University of Science and Technology, Kohat, Pakistan; 2 Department of Microbiology, Quaid-i-Azam University, Islamabad, Pakistan; 3 Department of Microbiology, Balochistan University of IT, Engineering and Management Sciences, Quetta, Pakistan; 4 Guizhou Key Laboratory of Microbiome and Infectious Disease Prevention and Control & Key Laboratory of Environmental Pollution Monitoring and Disease Control, Ministry of Education of Guizhou & School of Basic Medical Science & Institution of One Health Research, Guizhou Medical University, Gui'an New District, 561113, P.R. China; 5 FF Institute (Huzhou) Co., Ltd., Huzhou 313000, China; 6 State Key Laboratory of Microbial Metabolism, Joint International Research Laboratory of Metabolic & Developmental Sciences, School of Life Sciences and Biotechnology, Shanghai Jiao Tong University, Shanghai, China; 7 ASRT, Inc., Atlanta, Georgia, USA; 8 Department of Botany and Microbiology, College of Science, King Saud University, Riyadh 11451, Saudi Arabia

**Keywords:** *Alcaligenes pakistanensis*, GC-MS, molecular docking, extreme environments, passu glacier, cold-adapted

## Abstract

Antagonistic microorganisms from extreme environments have gained great attention from scientists due to increasing threat of global antimicrobial resistance. In this context, previously isolated cold-adapted isolate from Passu glacier *Alcaligenes pakistanensis* LTP10 was used to extract their antimicrobial metabolites with organic solvents. MIC and MBC assays of the extract were performed. The synergistic effect of LTP10 ethyl acetate extract was studied with known antibiotics against *Staphylococcus aureus* and *Escherichia coli*. The extract was analyzed by FTIR, GC-MS, and in silico studies. Ethyl acetate extract of LTP10 has shown a maximum activity of 20 ± 1.0 and 19.3 ± 1.2 mm against *Staphylococcus epidermidis* and *S. aureus*, respectively. MIC of the extract was 0.4 and 1.6 mg/mL against *S. aureus* and *E. coli*, respectively. FTIR analysis revealed the presesnce of functional groups belonging to alcohols, aliphatic hydrocarbons, and nitrogen containing organic compouns. GC-MS analysis confirmed the presence of important antimicrobial compounds Dodecanoic acid, 3-hydroxy-, 7,9-Di-tert-butyl-1-oxaspiro(4,5)deca-6,9-diene-2,8-dione, and Pyrimidine-2,4(1H,3H)-dione in ethyl acetate extract of LTP10. A molecular docking study of these compounds has shown that 7,9-Di-tert-butyl-1-oxaspiro(4,5)deca-6,9-diene-2,8-dione has strong binding affinity of -7.8 kcal/mol against dihydrofolate reductase. Moreover, an ADMET study of the compounds has predicted their good intestinal absorption and non-toxic nature. In this work, we uniquely identify and characterize antimicrobial metabolites of glacier-derived *Alcaligenes*, combining GC–MS profiling with in-silico analysis. It was concluded that *Alcaligenes pakistanensis* LTP10 could be considered a good source for antimicrobial compound production, which should be further characterized by analytical techniques.

## 1. Introduction

Extreme environments offer very low levels of nutrients and are known as oligotrophic environments. Microorganisms existing in such environments have extraordinary characteristics to produce different active metabolites [1]. Extremophiles are considered a new opportunity for exploring new biologically active metabolites and their applications in various fields [2]. Uncovering new antimicrobial compound reservoirs would be an essential part in the exposure of new drug candidates, rushing in development processes of drugs and mainly solving the problem of drug resistance to certain limits. Scientists are exploring a variety of extreme ecosystems, such as glaciers, deserts, hot springs, deep sea, mountains, and forests [3]. Our purpose of this study is to characterize the antimicrobial compounds in the crude extract of *Alcaligenes pakistanensis* LTP10 by GC-MS and an in silico study.

Earth is covered mostly by cold biospheres [4]. The cryosphere consists of large portions of glaciers and ice sheets on continents and comprise most of the Earth's freshwater. Glaciers cover almost 10% of the Earth, causing permanently cold environments [5],[6], mostly in the polar regions. Apart from the polar regions, the Hindu Kush, Karakoram, and Himalaya (HKKH) region comprises a major glacial reservoir and consist of almost 54,252 small and large glaciers covering an area of 60,000 km^2^. Thus, a number of scientists refer to it as a “third pole” [7]. Furthermore, psychrophilic microorganisms are the cold loving extremophiles [8] and are classified into two major types; one is considered true psychrophiles and the other is called psychrotrophs [9]. Psychrotrophs, or psychrotolerants, are present in the same icy ecosystems as psychrophiles but in larger quantities [4]. Scientists are extensively examining and pointing to unearth novel antimicrobial compounds from cold-adapted microorganisms because of their resistance to low temperatures and important applications in the medical field. Cold-adapted microorganisms exhibit potential for the production of antimicrobial compounds [10].

Humans extract many secondary metabolite secretions for their own health, to fill gaps in the food pyramid, to boost agricultural productivity, to have a lasting impact on the economy, and as a source of antibiotics [11]. The primary suppliers of secondary metabolites include several marine creatures, bacteria, fungus, and plants [12]. Secondary metabolites produced by microorganisms have peculiar structures and low molecular masses. According to Demain, structurally varied metabolites exhibit a wide range of biological roles, including as antibacterial agents, enzyme inhibitors, antitumor, immunosuppressants, antiparasitic agents, stimulators of plant growth, herbicides, insecticides, and anti-helmintics [13]. They are created when the bacteria are in their late development phase. The synthesis of secondary metabolites in microorganisms is regulated by specific regulatory mechanisms because it is often suppressed in the logarithmic phase and decreased in stationary growth stages [14].

Gas chromatography mass spectrometry (GC-MS) has solidified its position as a major technical platform for secondary metabolite profiling in plants and microorganisms during the past few years [15]. Because of its great separation power and ability to accurately identify hundreds of organic compounds in a single study, gas chromatography combined with MS (GC-MS) has been widely utilized in metabolomics [16],[17]. GC-MS systems are stable, strong, affordable, highly sensitive, and simple to operate. Additionally, the utilization of chemical libraries for the identification of metabolites is made possible by the very repeatable mass spectra produced by the electron impact ionization employed by typical GCMS equipment [18]. GC-MS instruments, however, examine only volatile chemicals [19].

The advancement of bioinformatics tools and services has a great advantage of screening many compounds for their pharmacokinetic properties and predicting their potential affinity toward drug targets [20]. For this purpose, a molecular docking study [21],[22] was accomplished to support antibacterial activity and to recognize the binding mechanism of the interactions of the selected antimicrobial compounds of *Alcaligenes pakistanensis* LTP10 against the binding site of the target enzymes DNA gyrase and dihydrofolate reductase. Moreover, the in silico ADMET (absorption, distribution, metabolic, excretion, toxicity) properties and drug-likeness [23] of the antimicrobial metabolites were also predicted to determine their bioavailability and toxicity.

## 2. Materials and methods

### 2.1. Description of isolate *Alcaligenes pakistanensis* LTP10

*Alcaligenes pakistanensis* LTP10 was isolated by the same research group from Passu glacier [24] in Karakoram Mountain range of Pakistan. The preserved culture of the bacterial isolate LTP10, in glycerol, was available at the Applied Environmental and Geomicrobiology Lab at Quaid-i-Azam University, Islamabad. To use the isolate, an aliquot of 200 µL of glycerol preserved bacterial culture was spread on a LB agar plate and incubated for 5 days at 4 °C. After incubation, the pure isolated colony of LTP10 was taken and processed for downstream application.

### 2.2. Optimization of solvent extraction from metabolites

For the extraction of antimicrobial products from *Alcaligenes pakistanensis* LTP10, the solvent phase extraction method was used. Organic solvents, including ethyl acetate, chloroform, and hexane, were studied for antimicrobial metabolite extraction from the supernatant. A 200 µL aliquot of glycerol preserved bacterial culture of *Alcaligenes pakistanensis* LTP10 was spread on an LB agar plate and incubated for 5 days at 4 °C. The culture was then grown in LB broth. A total of 1000 mL culture broth of LTP10 was subjected to centrifugation, and each 200 mL of CFS was then mixed separately with equal volumes of n-hexane, ethyl acetate, and chloroform and agitated thoroughly. A separating funnel was used to separate two immiscible layers, and the upper layer containing bioactive compounds was collected. This process was repeated 3 times. Thereafter, all the extracts of LTP10 were dried in a rotatory evaporator Buchi R-200 Rotavapor System, and dried crude powders were obtained. Then, using an agar well diffusion experiment, the antibacterial activity of the dried powder from each solvent extract, dissolved in non-inhibitory concentrations of dimethyl sulfoxide (DMSO), was evaluated. However, the agar diffusion method provided only the qualitative assessment of antibacterial activity. The extraction of crude extract was continued using the ethyl acetate extract, which demonstrated the observable activity in terms of zone of inhibition (ZOI). The positive control was a disc of amoxicillin (10 µg), while the negative controls were n-hexane, ethyl acetate, and chloroform.

### 2.3. MIC and MBC assay against ATCC strains

To determine MIC of ethyl acetate extract of *Alcaligenes pakistanensis* LTP10, ATCC test strains *Staphylococcus aureus* (ATCC 25923), *Salmonella enterica* (ATCC 14028), *Pseudomonas aeruginosa* (ATCC 27853), *Bacillus subtilis* (ATCC 6633), *Escherichia coli* (ATCC 25922), *and Staphylococcus epidermidis* (ATCC 12228) were utilized. To create single colonies, the test-relevant bacterial isolates (together with a control organism) were spread onto MHA plates without inhibitors. The plates were incubated overnight at 37°C. A single colony from the agar plates for each isolate was chosen, transferred to a conical flask with 50 mL MH-Broth, and cultured for 24 hrs at 37 °C at 150 rpm. Then, the OD_600_ of these cultures were measured, and dilutions were made in 5 mL MHB to OD_600_ = 0.01. MIC was assessed by the microdilution technique using 96 well microtiter plate [25],[26]. A total of 50 µL of Muller-Hinton broth was poured into the wells in the first 6 rows of columns 2–11. Then, MHB (100 µL) was poured into column 12, and 100 µL (12.8 mg/mL) of the crude extract was added to column 1. An aliquot of 50 µL was withdrawn from each well in column 1 and mixed with the subsequent wells in the 2^nd^ column. The process was repeated for columns 2 and 3, columns 3 and 4, up to column 10. The withdrawn solution from column 10 was discarded. The bacterial suspensions in MHB were vortexed, and 50 µL of each bacterial culture was poured into each well in single row in columns 1–11. The plates were covered with film to create a tight seal to prevent any evaporation. Each assay was performed in triplicate, and if the value was repeated at least two times, it was considered the MIC. The plates were kept at 37 °C in incubator for 16–24 hrs. The turbidity of the bacterial cultures was measured by determining OD_600_. The concentration of extract in mg/mL was considered the minimum inhibitory concentration, which resulted in minimum growth. After a 96-well plate incubation, dilutions that showed no sign of growth were streaked with a sterile wire loop on sterilized MHA plates. After that, the plates underwent another 24 hours of incubation. After the incubation time, the plates were inspected for bacterial growth. The lowest concentration that failed to form even a single colony was identified as the MBC value. The MIC and MBC values of ampicillin were included as reference control values obtained from CLSI guidelines [27].

### 2.4. Combined antibacterial activity of LTP10 extract with antibiotics

The antimicrobial activity of ethyl acetate extract of *Alcaligenes pakistanensis* LTP10 was estimated in combination with known antibiotics to assess their combined antibacterial activity. For this purpose, the disk diffusion method was used as a preliminary screening method for antibacterial activity and combination effects, in which sterile MHA was added to autoclaved petri dishes and then inoculated with *S. aureus* and *E. coli*. Inoculated MHA plates were exposed to the antibiotic discs (6 mm) of imipenem (10 µg), nalidixic acid (30 µg), penicillin (10 µg), and cefepime (30 µg). Antibiotics were selected as representatives of different mechanistic classes for in vitro comparative screening purposes. To evaluate the combined antibacterial activity of the crude extract and antibiotics, a second set of the same antibiotic discs were impregnated with 5 µL of crude extract (5 mg/mL), and they were placed on the inoculated agar surface. Before being incubated for 24 hrs at 37 °C, the petri plates were kept at ambient temperature for 30 minutes. Formation of a clear circular ZOI surrounding the discs, which corresponded to the lack of bacterial growth, was indicative of the extract working [28]. The inhibitory zone's diameter was measured in millimeters. Moreover, the diameter of the inhibition zone was a qualitative indicator of antimicrobial activity, influenced by diffusion characteristics and the molecular size of compounds.

### 2.5. GC-MS analysis of extract

The gas chromatograph (GC) used for the GC analysis had a capillary column and a detector (FID). The GC-MS evaluation of the ethyl acetate LTP10 extract was accomplished using a Shimadzu GC-17A fitted with a Shimadzu GCMSQP5050A mass selective detector and an HP-5 MS capillary column having dimensions of 30 m × 0.25 mm and film thickness of 0.25 mm. For GC-MS finding, electron ionization apparatus (EIA) having ionization energy of 70 eV was utilized. The carrier gas (helium) was set flowing at 0.8 mL/min rate. The mass spectrometer transfer line and injector were set to 270 °C and 250 °C, respectively. The oven temperature was maintained at 50 °C for three minutes before being raised by 3 °C/min to 240 °C. By comparing each component's average peak area to the total areas, the relative percentage amount of each component was calculated. Utilizing NIST library spectral data, compounds were tentatively identified, and the fragmentation pattern of mass spectra was compared to information that had been published in the literature [29],[30].

### 2.6. FTIR analysis of extract

To distinguish functional groups and chemical bonds in compounds, Fourier transform infrared spectrophotometer (FTIR) is considered the best technique [31]. Thus, Infra red (IR) analysis was performed using the PerkinElmer Spectrum 65 FT-IR Spectrometer. A dried powder of the LTP10 ethylacetate extract was used for the FTIR examination. A small amount of LTP10 extract dried powder was transferred to diamond crystal in the middle of a circular metallic plate. A movable pressure arm was then rotated to place it over the sample. IR rays were passed from below through sample and the spectrum was obtained. An FTIR spectrum at a frequency of 4 cm^−1^ with a scanning range of 515 to 4000 cm^−1^ was used to analyze the powdered sample.

### 2.7. Preliminary in-silico screening

The SMILES of putative compounds by GCMS were obtained from an online dataset at PubChem [32] and initially screened by druglikeness filters (Lipinski, Veber, PAINS) and safety filters (toxicity prediction (TOX), Rapid Elimination of Swill (REOS), human Ether-à-go-go-Related Gene (HERG)). Metabolites that were obtained after filtering were subjected to QSAR analysis to predict their pIC50 against bacterial targets DNA gyrase and dihydrofolate reductase from *S. aureus* and *E. coli*. For this purpose, IC50 data against gyrase and dihydrofolate reductase was retrieved from the ChEMBL database [33] and used to train models Random Forest and Linear regression. Metabolites were also subjected to batch docking by Autodock Vina [34] against the gyrase B subunit and dihydrofolate reductase. For batch docking, target proteins' pdb files were downloaded from the protein data bank RCSB [35],[36], and compound sdf files were retrieved from PubChem. Only 2 compounds were further selected for detailed docking and ADMET studies based on best docking score, which were pIC50 and reported antimicrobial activity.

### 2.8. Molecular docking study of important compounds

Molecular docking has become a major tool in computer-based drug designs to predict the binding affinity of ligand with target proteins and their interactions [37]. Molecular docking was used for important antimicrobial metabolites for GCMS analysis. These compounds were docked against the target proteins of test organisms *S. aureus* and *E. coli* using Autodock Vina. DNA gyrase and dihydrofolate reductase were selected as molecular targets due to their essential roles in bacterial survival, established validation as antibacterial drug targets, and availability of high-resolution crystal structures from the same test organisms. 3D structures of target proteins DNA gyrase (PDB ID: 5Z9P) and dihydrofolate reductase (PDB ID: 7NAE) were obtained from Protein Data Bank RSCB. PDB 5Z9P has a resolution of 1.45 A and is bound with a chemical fragment reported as an inhibitor [38], while PDB 7NAE has a resolution of 2.35 A and is bound with trimethoprim as an inhibitor [39]. The proteins were prepared for docking by removing their water molecules and ligand molecules using BIOVIA Discovery Studio software (2024 version), and hydrogen and kollman charges were added using Autodock tools [40] and saved as pdbqt files. The gridbox was calculated with Autodock tools according to the target site with the following dimensions and spacing: center_x = 146.198 Å, center_y = -3.523 Å, center_z = 63.708 Å, size center_x = 32.594 Å, size center_y = 42.163 Å, and size center_z = 37.269 Å for 5Z9P and center_x = -18.729 Å, center_y = 21.115 Å, center_z = -1.223 Å, size center_x = 34.613 Å, size center_y = 35.383 Å, and size center_z = 31.749 Å for 7NAE. To validate the docking protocol, the original inhibitors (co-crystallized ligands) were removed from both target proteins and saved as pdb files using Discovery studio. The inhibitors trimethoprim and chemical fragment were protonated using Open Babel [41] at pH 7 and 8, respectively, and then redocked with their target proteins, and PyMOL (2.5.4 version) was used to calculate their RMSD [42]. Two compounds, 7,9-Di-tert-butyl-1-oxaspiro (4,5) deca-6,9-diene-2,8-dione and Dodecanoic acid, 3-hydroxy-, were selected for a detailed docking study. However, for compound Dodecanoic acid, 3-hydroxy-, its two enantiomers (R)-3-hydroxydodecanoic acid and (S)-3-hydroxydodecanoic acid were used separately for docking, as the stereochemistry at C-3 of Dodecanoic acid, 3-hydroxy- was not defined in the GC–MS identification. Accordingly, 3D structures of these compounds were obtained from the PubChem database. The compounds were prepared for docking by protonation at pH 7 and 8, adding polar hydrogens and Gasteiger charge using Autodock tools and saved as pdbqt files. The compounds were then docked against the proteins gyrase and dihydrofolate reductase by Autodock Vina to determine their binding affinity. The results of Autodock Vina were visualized in PyMOL and Discovery studio. Bond lengths were calculated in PyMOL using the Measurement wizard tool, which displayed distances between donor and acceptor atoms angstroms (Å). Measurements below 3.5 Å were considered strong.

### 2.9. ADMET and drug-likeness prediction of compounds

In silico prediction of absorption, distribution, metabolism, excretion, and toxicity (ADMET) of compounds has become an important part of pharmaceutical research and development [43]. Online web servers ADMETlab 3.0 [44] and pkCSM [45] were used to predict the ADMET properties of two important metabolites, 7,9-Di-tert-butyl-1-oxaspiro (4,5) deca-6,9-diene-2,8-dione and Dodecanoic acid, 3-hydroxy-, and one reference compound, trimethoprim. For this purpose, the canonical SMILES of the compounds were obtained from the PubChem database and analyzed by the webservers. Similarly, their drug likeness was predicted using webtools SwissADME [46] and ADMETlab 3.0. The compounds were assessed by Lipinski's Rule of Five, Veber's rule, Ghose filter, bioavailability score, synthetic accessibility score, PAINS filter, and Brenk filter.

### 2.10. Statistical analysis

By calculating the standard deviation (stdev) and p-value, the difference in activity under various cultural situations was examined by statistical analysis. For the purpose of calculating the statistical importance of variations in ZOI under various culture circumstances, the p-value was computed using T.Test in Microsoft Excel 365 [47]. Each experiment was run three times. Mean ± SD (n + 3) was used to depict the values. The significance threshold was p < 0.05, and the confidence level was 95%.

## 3. Results

### 3.1. Extraction of antimicrobial compounds

**Figure 1.**
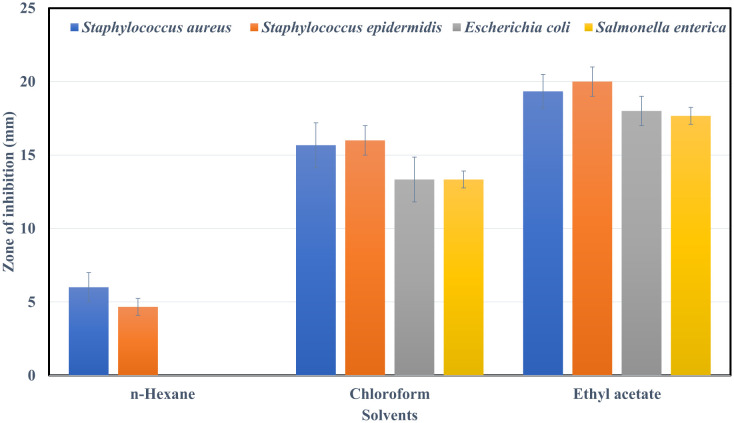
Antibacterial activity of extracts of *A. pakistanensis* LTP10 in various solvents measured in mm (ZOI) against ATCC test organisms. The error bars denote the SD of triplicate ZOI measurements. The p-value is less than 0.05.

The solvents used for the extraction of antimicrobial metabolites from CFS of ethyl acetate extract of *Alcaligenes pakistanensis* LTP10 were n-hexane, ethyl acetate, and chloroform. After the extraction process, the extracts of different solvents were assessed for antagonistic activity by agar well diffusion assay, as shown in Figure 1. The results showed that ethyl acetate extract has the largest zones of inhibition (mm) among the tested extracts against ATCC test strains *S. aureus, E. coli, S. epidermidis*, and *S. enterica* followed by chloroform. The difference between the activity of ethyl acetate extract and chloroform extract was statistically significant, as was evident from the p-value (P < 0.05). However, n-hexane showed no or little activity against the test strains. Based on these results, ethyl acetate was further used for the extraction of antimicrobial metabolites from CFS of *Alcaligenes pakistanensis* (LTP10). Moreover, the positive control amoxicillin (10 µg) showed 23–30 mm ZOI against the tested strains, while the negative controls, hexane, ethyl acetate, and chloroform, did not produce any zones against the tested strains.

### 3.2. MIC and MBC assay

MIC of the extract was determined against ATCC test strains. The MIC of *Alcaligenes pakistanensis* LTP10 extract against *S. aureus* was 0.4 mg/mL and against *E. coli*, it was 1.6 mg/mL. Similarly, MIC against other bacteria (*B. subtilis, S. epidermidis, P. aeruginosa*, and *S. enterica*) were 0.8, 0.8, 3.2, and 1.6 mg/mL, respectively, as shown in Table 1. To observe the bactericidal effect of ethyl acetate extract of LTP10, the dilutions in the MIC test, which did not show any growth, were used. It was observed that *S. aureus* showed no growth from any of the dilutions, and its MBC was the same as MIC; i.e., 0.4 mg/mL. Similarly, the MBC values of the LTP10 extract against *B. subtilis* and *S. epidermidis* was 1.6 mg/mL. However, *E. coli, P. aeruginosa*, and *S. enterica* showed growth in all dilutions, indicating non-bactericidal behaviour of the LTP10 extract against these microorganisms. In comparison, ampicillin exhibited much lower MIC values (0.3–4 µg/mL) against most test strains, suggesting higher potency. However, *P. aeruginosa* was resistant to ampicillin (MIC ≥64 µg/mL), while the extract demonstrated considerable inhibitory activity (MIC 3.2 mg/mL).

**Table 1.** MIC and MBC values of ethyl acetate extract of LTP10 against test strains.

Test organisms	Extract		Ampicillin (Control)	
	MIC (mg/mL)	MBC (mg/mL)	MIC (µg/mL)*	MBC (µg/mL)*
Staphylococcus aureus	0.4	0.4	1	2
Escherichia coli	1.6	--	4	8
Bacillus subtilis	0.8	3.2	0.3	1
Staphylococcus epidermidis	0.8	3.2	1	4
Pseudomonas aeruginosa	3.2	---	≥64 (resistant)	≥128
Salmonella enterica	1.6	---	4	8

* MIC and MBC values of ampicillin are based on CLSI (2020) guidelines and are included for reference only; these values were not experimentally determined.

### 3.3. Combined antibacterial activity of LTP10 extract with antibiotics

**Figure 2.**
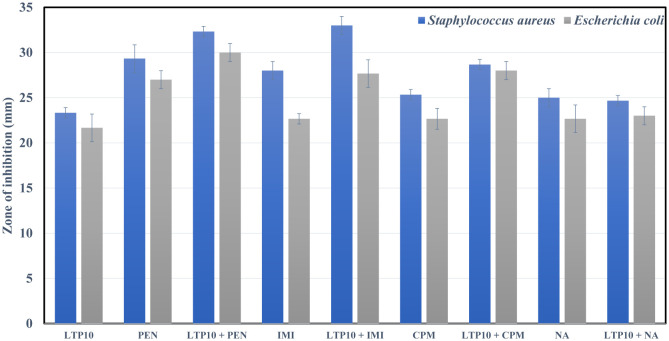
Antibacterial activity of ethyl acetate extract of *Alcaligenes pakistanensis* LTP10 with and without antibiotics measured in mm (ZOI) against *S. aureus* and *E. coli*. The error bars denote the SD of triplicate ZOI measurements. The p-value was less than 0.05. PEN; Penicillin, IMI; Imipenem, CPM; Cefepime, and NA; Nalidixic acid.

The ethyl acetate extract of LTP10 was evaluated for its combined antibacterial activity with known antibiotics (penicillin, imipenem, cefepime, nalidixic acid) against test strains *S. aureus* and *E. coli*. As shown in Figure 2, there was an increase in the size of ZOI of LTP10 extract (5 mg/mL) when combined with penicillin 10 µg, imipenem 10 µg, and cefepime 30 µg. However, there was no or little increase in ZOI of LTP10 when combined with nalidixic acid 30 µg. These results suggested a possible interaction of LTP10 with antibiotics. The difference in ZOI of LTP10 extract alone and combined with antibiotic discs (penicillin, imipenem, cefepime) was statistically significant, as evident from the p-value (P < 0.05).

### 3.4. GC-MS analysis of extract

**Figure 3.**
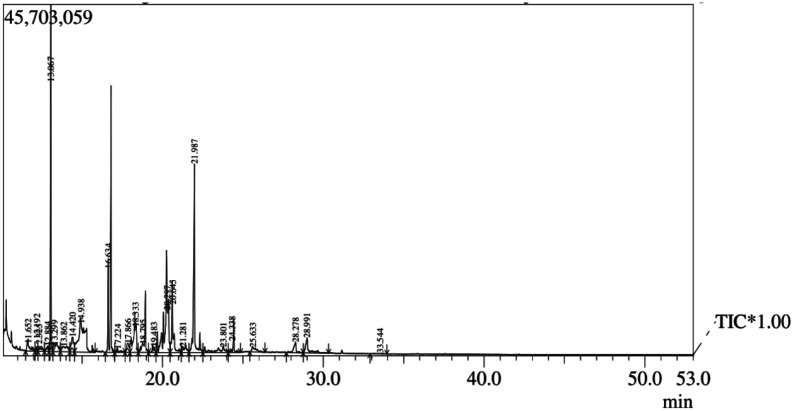
GC/MS chromatogram of ethyl acetate extract of *Alcaligenes pakistanensis* LTP10.

Gas chromatography/mass spectroscopy was performed for the evaluation of the ethyl acetate extract of LTP10. The GC-MS analysis showed the presence of more than 30 peaks, indicating the presence of microbial compounds, as shown in Figure 3. The mass spectra obtained was automatically compared with the NIST database, and 22 metabolites were tentatively characterized and recognized as indicated by their similarity index (Table 2). Some of the important metabolites were 2,4-Di-tert-butylphenol (Cpd 4), Dodecanoic acid, 3-hydroxy- (Cpd 10), 7,9-Di-tert-butyl-1-oxaspiro(4,5) deca-6,9-diene-2,8-dione (Cpd 13), Pentadecanoic acid,14-methyl-,methyl ester (Cpd 8), Pyrrolo[1,2-a]pyrazine-1,4-dione, hexahydro-3-(2-methylpropyl) (Cpd 12), 1-Nonadecanol (Cpd 17), Oleic Acid (Cpd 20), and Heptadecanoic acid, hepta-decylester (Cpd 22), with reported bioactivities, like antibacterial, antifungal, and anticancer activities.

**Table 2.** Details of tentatively identified compounds in ethyl acetate extract of *Alcaligenes pakistanensis* LTP10.

Cpd	Formula	Area (%)	RT (min)	Tentative compound	Activity reported	Similarity Index (SI)	Reference
1	C_6_H_9_NO_3_	1.55	11.625	DL-Proline, 5-oxo-, methyl ester	antitumor	89	[48]
2	C_9_H_18_O	0.71	12.192	trans-3,4-Epoxynonane	not reported	75	NA
3	C_5_H_12_N_2_O_2_S	0.6	12.884	S-[2-Aminoethyl]-dl-cysteine	antibacterial	65	NA
4	C_14_H_22_O	11.27	13.067	2,4-Di-tert-butylphenol	antifungal antibacterial	90	[49]
5	C_9_H_19_NO	2.09	13.299	Azacyclodecan-5-ol	not reported	71	NA
6	C_11_H_22_	2.18	14.420	2-Octene, 2,3,7-trimethyl	not reported	77	NA
7	C_12_H_16_N_4_O_5_	11.65	14.938	Pyrimidine-2,4(1H,3H)-dione	antimicrobial	61	[50]
8	C_17_H_34_O_2_	11.9	16.634	Pentadecanoic acid, 14-methyl-, methyl ester	antibacterial antifungal	94	[51]
9	C_28_H_56_O_2_	0.58	17.224	Heptacosanoic acid, methyl ester	not reported	84	NA
10	C_12_H_24_O_3_	1.31	17.866	Dodecanoic acid, 3-hydroxy-	antifungal antibacterial	81	[52]
11	C_9_H_14_O_2_	6.12	18.333	6-Oxabicyclo[3.1.0]hexan-3-one	not reported	78	NA
12	C_11_H_18_N_2_O_2_	4.13	18.795	Pyrrolo[1,2-a]pyrazine-1,4-dione, hexahydro-3-(2-methylpropyl)	antioxidant	80	[53]
13	C_17_H_24_O_3_	0.89	19.483	7,9-Di-tert-butyl-1-oxaspiro (4,5) deca-6,9-diene-2,8-dione	antimicrobial	74	[54]
14	C_22_H_44_O_2_	14.98	20.297	Heneicosanoic acid, methyl ester	not reported	84	NA
15	C_18_H_34_O_2_	5.32	20.645	Cyclopropaneoctanoic acid, 2-hexyl-, methyl ester	not reported	90	NA
16	C_15_H_30_O_2_	1.48	21.281	Pentadecanoic acid	antioxidant	79	[55]
17	C_19_H_40_O	11.51	21.987	1-Nonadecanol	antibacterial	96	[56]
18	C_11_H_16_O_4_	2.49	23.801	9,9-Dimethoxybicyclo [3.3.1]nona-2,4-dione	cytotoxic	81	[57]
19	C_17_H_32_O_2_	1.83	24.338	7-Hexadecenoic acid, methyl ester, (Z)-	antimicrobial antioxidant	88	[58]
20	C_18_H_34_O_2_	1.17	25.633	Oleic Acid	antibacterial	89	[59]
21	C_14_H_16_N_2_O_2_	1.48	28.278	Pyrrolo[1,2-a]pyrazine-1,4-dione, hexahydro-3-(phenylmethyl)	antifungal	77	[60]
22	C_34_H_68_O_2_	0.1	33.544	Heptadecanoic acid, heptadecyl ester	antibacterial	80	[61]

### 3.5. Interpretation of mass spectral fragmentation

**Figure 4.**
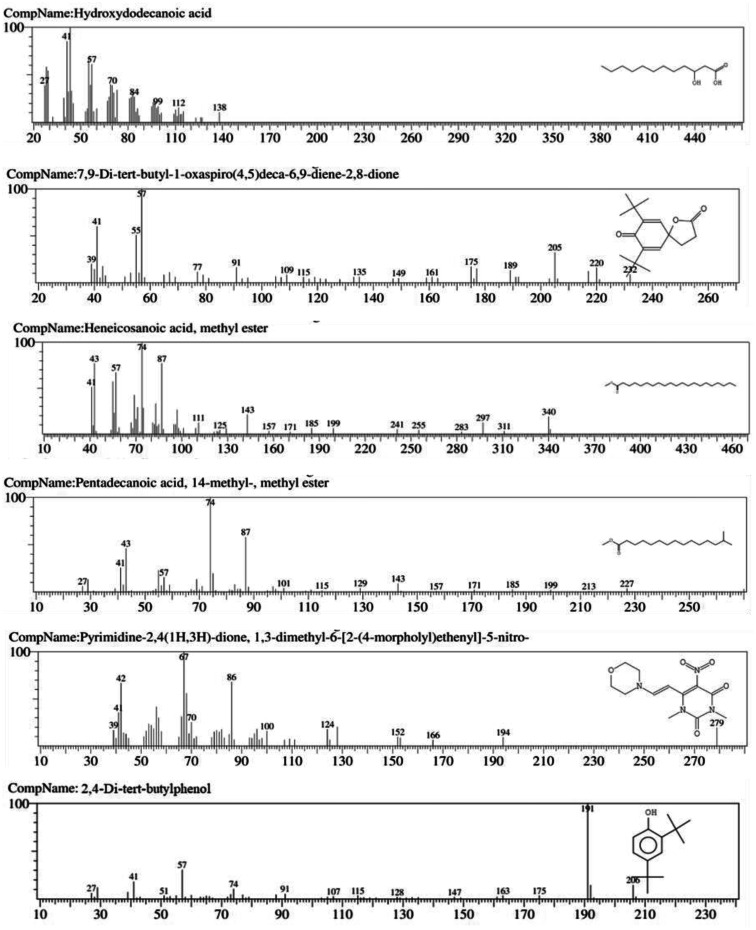
Mass spectra and chemical structures of important compounds identified in ethyl acetate extract of *Alcaligenes pakistanensis* LTP10.

The mass spectra and chemical structures of important compounds are shown in Figure 4. These spectra have shown specific fragmentation patterns coherent with their proposed structures. Dodecanoic acid, 3-hydroxy- exhibited classic fatty acid fragmentation with prominent ions at m/z values 41, 57, 70, corresponding to alkyl chain cleavage and beta-cleavage adjacent to the hydroxyl group. The compounds pentadecanoic acid, 14-methyl-, methyl ester, and heneicosanoic acid demonstrated prominent signals at m/z 43 and 74, which are characteristic of methyl esters. the presence of signals at m/z values 57. 175, 189, and 205 in the mass spectrum of the compound 7,9-di-tert-butyl-1-oxaspiro (4,5) deca-6,9-diene-2,8-dione could be assigned to cleavage of tert-butyl groups and stabilization of the spirodione ring system. The presence of base at m/z 191 in mass spectrum of 2,4-Di-tert-butylphenol indicated the loss of a methyl group from tert-butyl substituents, along with typical aromatic ring stabilization fragments. The fragmentation pattern of pyrimidine-2,4(1H,3H)-dione was more complex with prominent peaks at m/z 67 and 86, representing cleavage of heterocyclic ring and side-chain substituents. Overall, the fragmentation patterns observed in the mass spectra of compounds support their tentative identification based on GC-MS analysis.

### 3.6. FTIR analysis of extract

**Figure 5.**
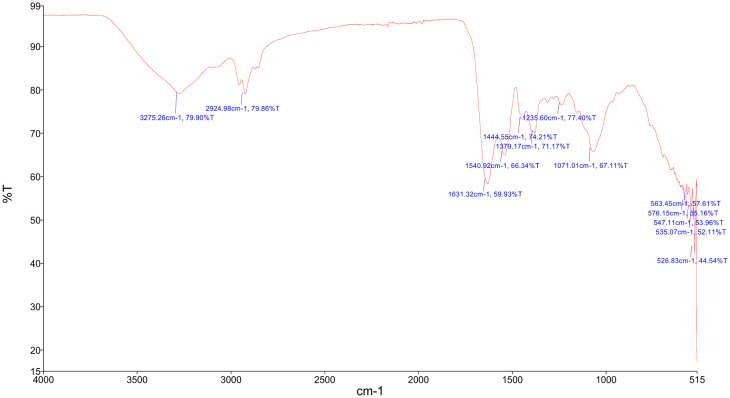
FTIR spectrum of ethyl acetate extract of *Alcaligenes pakistanensis* LTP10.

Ethyl acetate extract was exposed to IR radiations in the FTIR instrument, resulting in the identification of metabolites' functional groups due to absorption of different IR radiations. The analysis of the FTIR spectrum showed the existence of important peaks at 3275, 2924, 1631, 1540, 1444, 1380, 1235, and 1071 cm^−1^ corresponding to O-H, C-H, C=C, C-O, N-O, S=O, C-N, and C-O functional groups, respectively, as shown in Figure 5 and Table 3.

**Table 3.** FTIR spectroscopic data of ethyl acetate extract of *Alcaligenes pakistanensis* LTP10 and their functional groups.

Frequency (cm^−1^)	Bond strength	Functional Group	Compound Class
3275	strong, broad	O-H stretching	alcohol
2924	medium	C-H stretching	alkane
1631	medium	C=C stretching	alkene
1540	strong	N-O stretching	nitro compound
1444	medium	C-H bending	alkane
1380	strong	S=O stretching	sulfate
1235	medium	C-N stretching	amine
1071	strong	C-O stretching	primary alcohol

### 3.7. Preliminary in-silico screening

Preliminary screening of 22 compounds from GCMS by drug likeness filters (Lipinski, Veber, PAINS) indicated 13 compounds fulfilling criteria, while safety filters TOX, REOS, and HERG further reduced the number to 9 non-toxic compounds, as shown in Table 4. The average pIC50 of the 9 compounds against the targets gyrase and dihydrofolate reductase, predicted by best performance model random forest, was further used in selecting the top 5 compounds Cpd 13, Cpd 18, Cpd 3, Cpd 5, and Cpd 10. Batch docking results showed binding affinities of top 5 compounds from -4.6 to -7.8 kcal/mol. Based on preliminary in-silico analysis and reported antimicrobial activity, the compounds 7,9-Di-tert-butyl-1-oxaspiro(4,5) deca-6,9-diene-2,8-dione (Cpd 13) and Dodecanoic acid, 3-hydroxy- (Cpd 10) were selected for further detailed molecular docking, ADMET, and drug likeness evaluation.

**Table 4.** Preliminary in silico analysis of GCMS identified metabolites of LTP10.

Cpd	Compound	Predicted Avg. pIC50	Binding Affinity (kcal/mol)	
			*GYR	**DHFR
13	7,9-Di-tert-butyl-1-oxaspiro (4,5)deca-6,9-diene-2,8-dione	5.13	-6.0	-7.8
18	9,9-Dimethoxybicyclo [3.3.1]nona-2,4-dione	4.73	-5.4	-6.6
3	S-[2-Aminoethyl]-dl-cysteine	4.63	-4.6	-4.6
5	Azacyclodecan-5-ol	4.62	-5.4	-6.1
10	Dodecanoic acid, 3-hydroxy-	4.54	-5.5	-5.7
6	2-Octene, 2,3,7-trimethyl	4.13	-5.8	-5.5
11	6-Oxabicyclo[3.1.0]hexan-3-one	3.95	-3.7	-3.7
7	Pyrimidine-2,4(1H,3H)-dione	3.77	-4.6	-4.7
2	trans-3,4-Epoxynonane	3.72	-4.7	-4.8

*GYR = DNA gyrase (5Z9P) enzyme form *S. aureus*; **DHFR = Dihydrofolate reductase (7NAE) from *E. coli*.

### 3.8. Molecular docking study

**Figure 6.**
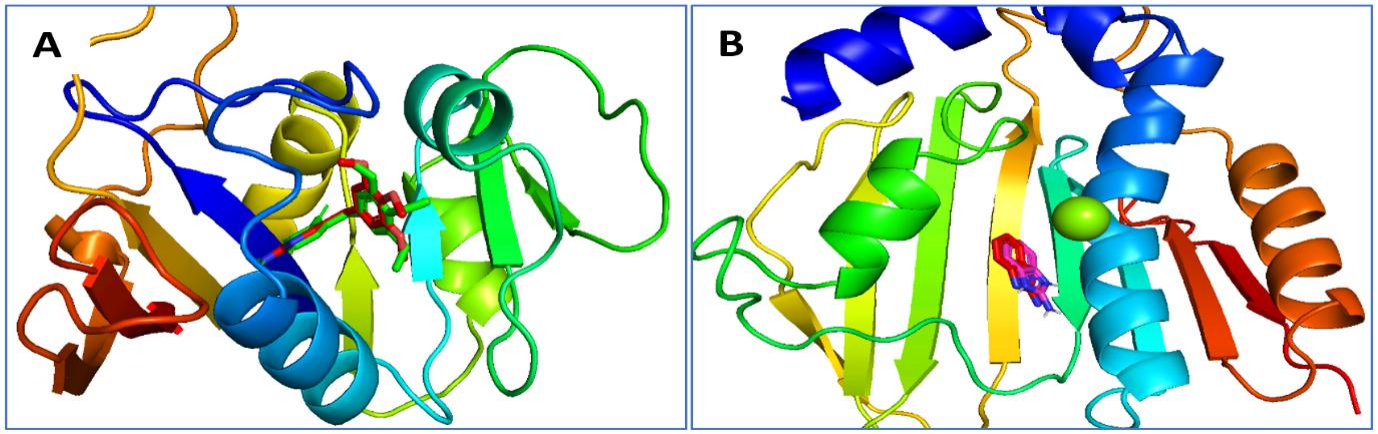
Redocking of original crystal ligands with their target proteins to validate the docking protocol. A) Redocked ligand trimethoprim with Dihydrofolate reductase (7NAE) of *Escherichia coli*. RMSD was 0.694 Å. B) Redocked ligand chemical fragment with DNA Gyrase B subunit (5Z9P) of *S. aureus*. RMSD was 1.113 Å.

**Figure 7.**
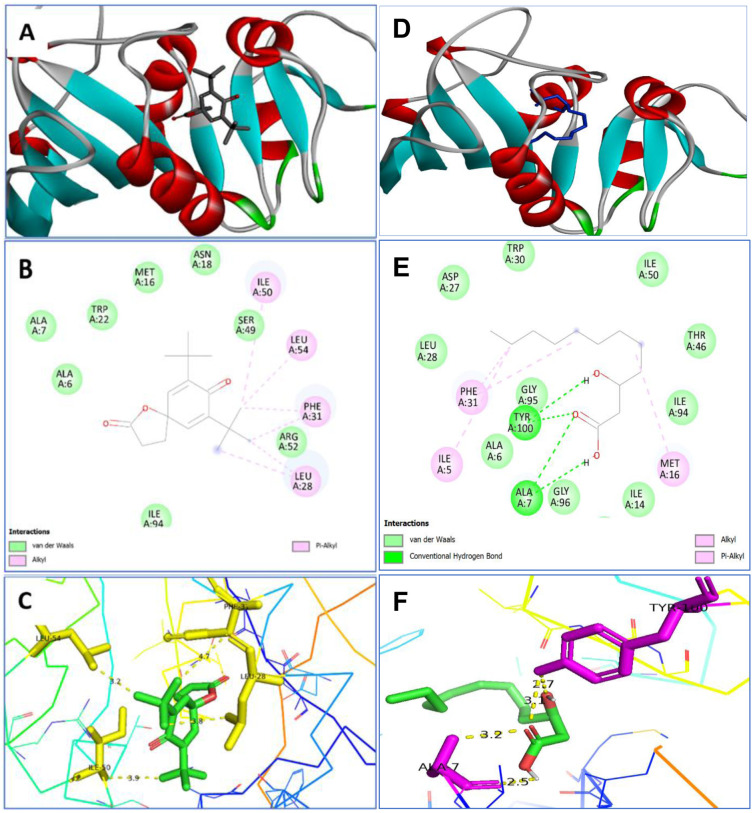
Molecular docking analysis of Dihydrofolate Reductase of *Escherichia coli* with 7,9-Di-tert-butyl-1-oxaspiro (4,5) deca-6,9-diene-2,8-dione (A, B, C) and (R)-3-Hydroxydodecanoic acid (D, E, F). 3D visualization of interactions (A & D), amino acid contact residues and types of interactions (B & E), and bond length in Å between the compound and binding site contact residues (C & F).

**Figure 8.**
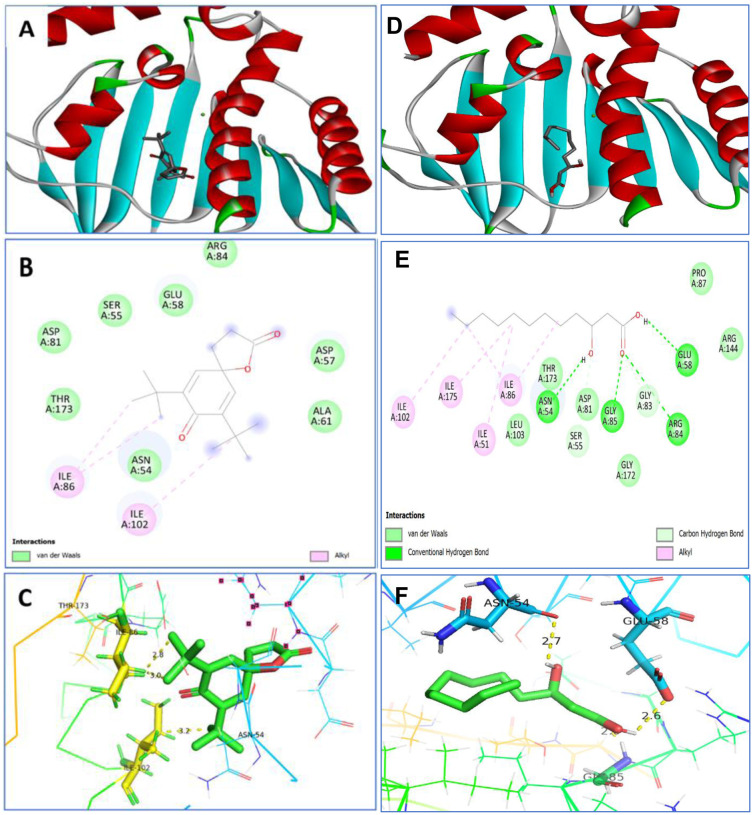
Molecular docking analysis of DNA Gyrase subunit B of *Staphylococcus aureus* with 7,9-Di-tert-butyl-1-oxaspiro (4,5) deca-6,9-diene-2,8-dione (A, B, C) and (R)-3-Hydroxydodecanoic acid (D, E, F). 3D visualization of interaction (A & D), amino acid contact residues and types of interactions (B & E), and bond length in Å between compound and binding site contact residues (C & F).

Redocking the co-crystallized ligands with their respective target proteins produced RMSD values below the accepted threshold of 2.0 Å, confirming the reliability of the docking protocol. RMSD of trimethoprim against 7NAE was 0.694 Å, and chemical fragment against 5Z9P showed RMSD 1.113 Å, as shown in Figure 6. The results of molecular docking for selected metabolites 7,9-Di-tert-butyl-1-oxaspiro (4,5) deca-6,9-diene-2,8-dione (Cpd 13), R and S enantiomers of Dodecanoic acid, 3-hydroxy- (Cpd 10), and original crystal ligands trimethoprim and chemical fragment, were obtained in the form of binding affinity, measured in kcal/mol, as given in Table 5. It was evident from the docking results that metabolite Cpd 13 showed a strong binding affinity of -7.8 kcal/mol against target 7NAE and -5.8 kcal/mol against 5Z9P. The experimental ligands also showed similar binding affinity against target proteins; i.e., -7.8 kcal/mol of trimethoprim against 7NAE and -6.0 kcal/mol of chemical fragment against 5Z9P. The same was evident from hydrogen and hydrophobic (alkyl, π-alkyl, and van der Waals) bonds at the site of ligand interactions with target enzymes, as shown in Figure 7. The contact residues of 7NAE with Cpd 13 were ILE50, LEU54, PHE31, and LEU28, and with trimethoprim were TYR100, ILE94, ILE50, and PHE31. Similarly, the contact residues of 5Z9P with Cpd 13 were ILE86 and ILE102, and with chemical fragment were ILE86, ASP81, and ASN54. Moreover, R and S enantiomers of compound Cpd 10 confirmed a binding affinity of -6.2 and -6.1 kcal/mol against target 7NAE and -5.8 and -5.5 kcal/mol against 5Z9P with contact residues TYR100 and ALA7 and GLU58, ARG84, and GLY85, respectively. The PyMOL interaction map shows that certain bond lengths of ligands Cpd 13 and Cpd 10 with interacting residues of target proteins were below 3.5 Å, suggesting strong interactions, as shown in Figure 7 (C, F) and Figure 8 (C, F). Overall, the results revealed good binding interactions of these metabolites in the ethyl acetate extract of LTP10 with bacterial target enzymes.

**Table 5.** Binding affinity, interaction bonds, and number of interacting bonds of target proteins Dihydrofolate Reductase from *E. coli* and DNA Gyrase B subunit from *S. aureus* with selected compounds.

Ligand	Target protein	PDB ID	Binding affinity (kcal/mol)	Contact residues	H-bonds	Hydrophobic interactions
7,9-Di-tert-butyl-1-oxaspiro (4,5) deca-6,9-diene-2,8-dione	Dihydrofolate Reductase	7NAE	-7.8	ILE50, LEU54, PHE31, LEU28	0	6
	DNA Gyrase B subunit	5Z9P	-5.8	ILE86, ILE102	0	3
(R)-3-Hydroxydodecanoic acid	Dihydrofolate Reductase	7NAE	-6.2	TYR100, ALA7, PHE31, ILE5	4	4
	DNA Gyrase B subunit	5Z9P	-5.8	GLU58, ARG84, GLY85, ASN54	4	5
(S)-3-Hydroxydodecanoic acid	Dihydrofolate Reductase	7NAE	-6.1	TYR100, ALA7, GLY15, GLY95	5	4
	DNA Gyrase B subunit	5Z9P	-5.5	GLU58, ARG84, GLY85, ILE102	3	6
Trimethoprim (Crystal ligand)	Dihydrofolate Reductase	7NAE	-7.8	TYR100, ILE94, ILE50, PHE31	4	8
Chemical fragment (Crystal ligand)	DNA Gyrase B subunit	5Z9P	-6.0	ILE86, ASP81, ASN54	1	2

### 3.9. Pharmacokinetic property prediction of compounds

An in silico prediction study of pharmacokinetic and toxicity properties of metabolites Cpd 13, Cpd 10 and reference trimethoprim (Trim) was performed using online webtools ADMETlab 3.0 and pkCSM, as shown in Table 6. Both compounds showed good human intestinal absorption, as predicted by both tools. None of the compounds were predicted as P-gp inhibitors, and only trimethoprim was classified as a P-gp substrate. Both the test compounds were found to have poor BBB permeability, while trimethoprim was predicted to have good CNS permeability. CYP inhibition demonstrated that neither test compounds nor reference compounds were likely to inhibit CYP3A4 and CYP2D6. Total clearance was acceptable for all 3 compounds. The non-mutagenic nature of all the three compounds was predicted by the AMES toxicity test and non-hERG inhibitory, indicating low cardiotoxicity risk. Hepatotoxicity result predictions indicated lower risk for microbial metabolites Cpd 13 and Cpd 10, while trimethoprim showed higher probability of hepatotoxicity. Rat acute toxicity (LD50) values showed that trimethoprim had the highest acute toxicity compared to microbial metabolites with low acute toxicity. Overall, the computational assessment indicated that both metabolites exhibited favorable pharmacokinetic and safety profiles in several cases superior to trimethoprim.

**Table 6.** Pharmacokinetic and toxicity properties prediction of metabolites Cpd10 and Cpd13 in ethyl acetate extract of *Alcaligenes pakistanensis* LTP10 and reference antibiotic trimethoprim (Trim).

Property	ADMETlab 3.0 (0-1, probability)*			pkCSM			pkCSM Units
	Cpd13	Cpd10	Trim	Cpd13	Cpd10	Trim	
Human Intestinal Absorption	0.276	0.700	0.000	98.22	91.39	79.51	%
Caco-2 permeability**	-4.556	-5.235	-4.951	1.400	1.438	0.606	log Papp
P-gp inhibitor	0.916	0.000	0.061	No	No	No	---
P-gp substrate	0.123	0.117	0.265	No	No	Yes	---
Volume of Distribution***	0.298	0.291	0.001	0.058	-0.934	0.193	log L/kg
BBB permeation	0.016	0.015	0.884	-0.045	0.145	-0.234	log BB
CYP2D6 inhibitor	0.008	0.001	0.000	No	No	No	---
CYP3A4 inhibitor	0.542	0.000	0.002	No	No	No	---
Total clearance****	7.834	4.115	6.381	0.798	1.663	0.381	log ml/min/kg
AMES mutagenicity	0.354	0.074	0.571	No	No	No	---
hERG inhibition	0.038	0.095	0.219	No	No	No	---
Hepatotoxicity	0.660	0.473	0.779	No	No	Yes	---
LD50 / acute toxicity (rat)	0.291	0.096	0.417	1.750	1.835	2.648	mol/kg
Skin sensitization	0.973	0.846	0.259	No	No	No	---

* Probability: 0-0.3: excellent; 0.3-0.7: medium; 0.7-1.0: poor ** > -5.15: excellent; otherwise: poor *** 0.04-20: excellent; otherwise: poor **** 0-5: excellent; 5-15: medium; > 15: poor

### 3.10. Drug-likeness

The results of drug likeness and medicinal chemistry filters demonstrated that compounds Cpd 13 and Cpd 10 along with reference trimethoprim fully complied with the Lipinski rule of five, Veber's rule, and Ghose filter, as shown in Table 7, indicating their strong potential for oral bioavailability and acceptable physicochemical characteristics. A bioavailability score of 0.85 for Cpd 10 indicated its higher systemic availability after oral absorption compared to Cpd13 (0.55) and trimethoprim (0.55). The synthetic accessibility score of all 3 compounds demonstrated their easy synthesis. No alerts by either PAINS or Brenk filters predicted that all the compounds showed no significant red flags related to safety.

**Table 7.** Drug-likeness and synthetic accessibility assessment of compounds Cpd13, Cpd10, and Trimethoprim by SwissADME and ADMETlab 3.0 using Lipinski's, Veber, Ghose, PAINS, and Brenk filters, as well as bioavailability and synthetic accessibility scores.

Tool	Property	Cpd13	Cpd10	Trim
SwissADME	Lipinski's Rule of Five	Yes (0 violations)	Yes (0 violations)	Yes (0 violations)
	Veber's Rule (TPSA, RotB)	Yes	Yes	Yes
	Ghose Filter	Yes	Yes	Yes
	Bioavailability Score	0.55	0.85	0.55
	Synthetic Accessibility Score	4.35 (medium)	2.67 (easy)	2.58 (easy)
	PAINS Filter	0 alert	0 alert	0 alert
	Brenk Filter	0 alert	0 alert	0 alert
ADMETlab 3.0	Lipinski's Rule of Five	Yes (0 violations)	Yes (0 violations)	Yes (0 violations)
	Synthetic Accessibility Score	3.0 (easy)	2.0 (easy)	2.0 (easy)
	PAINS Filter	0 alert	0 alert	0 alert

Bioavailability Score: 0–1, higher = better oral bioavailability Synthetic Accessibility Score: 1 = very easy, 10 = very difficult

## 4. Discussion

Earth is covered mostly by cold biosphere [4]. The psychrophilic environments present in polar regions, including Antarctica and Greenland, are widely examined for microbe variety and environmental and biogeochemical processes. In contrast, less interest has been given to non-polar psychrophilic environments, including the Karakoram Mountain region, which is a major glaciated area beyond the polar region. Moreover, the momentum to discover a third pole for microbial diversity and to search for novel bioactive compounds has increased [7],[62]. Additional motivation to investigate this topic is provided by the development of antimicrobial substances from other sources in response to antibiotic resistance and drug resistance [10]. These microorganisms need a wide range of biochemical and physiological modifications in order to live under the continual effect of low temperatures, strong winds, limited nutrients, and intense UV radiation, or combinations of these characteristics [63]. *Alcaligenes* strains from glacier environments are repeatedly reported to produce antibacterial metabolites; however, studies have lacked detailed chemical identification and mechanistic insights. In this study, *Alcaligenes pakistanensis* LTP10 was found to produce multiple antimicrobial metabolites, as confirmed by GCMS and in-silico analysis. These findings provide new structural and functional insights into glacier-derived *Alcaligenes*, addressing gaps in research.

A liquid-liquid separation technique is used to separate metabolites of interest into an immiscible solvent [64]. For this purpose, the CFS of LTP10 was extracted with n-Hexane, ethyl acetate, and chloroform, followed by their antimicrobial evaluation. The results indicated that ethyl acetate has good antimicrobial activity against *S. aureus, S. epidermidis, E. coli*, and *S. enterica*, followed by chloroform extract. However, n-Hexane showed no or little antimicrobial activity. Marrez et al. reported the use of solvents, including hexane, chloroform, and ethyl acetate, to extract antimicrobial metabolites of *Scenedesmus obliquus* and evaluated their antimicrobial activity by the disc diffusion method [65]. Khim et al. evaluated the use of hexane, diethyl ether, chloroform, ethyl acetate, acetone, and methanol for antimicrobial compound extraction [66]. Bose et al. described the use of ethyl acetate to extract antibiotics from culture of obligate marine actinobacterial species *Salinispora arenicola* [67]. Kapley et al. reported that ethyl acetate extract of *Alcaligenes* sp. HPC 1271 has clear ZOI against gram negative and gram positive microbes [68].

MIC and MBC of ethyl acetate extract of *Alcaligenes pakistanensis* LTP10 was evaluated against ATCC test strains. MIC of the extract was in the range of 0.4 to 3.2 mg/mL for different strains. The lowest MIC (0.4 mg/mL) was against *S. aureus*, while the highest MIC (3.2 mg/mL) was against *P. aeruginosa*. MBC of extract was in the range of 0.4 to 3.2 mg/mL, with the lowest MBC of 0.4 mg/mL against *S. aureus* and highest MBC 3.2 mg/mL against *S. epidermidis* and *B. subtilis*. However, *E. coli, P. aeruginosa*, and *S. enterica* showed growth even after treatment with the highest concentraion of extact (12.8 mg/mL). MIC and MBC against *S. aureus* were the same (0.4 mg/mL), while MBC was three folds greater for *S. epidermidis* and *B. subtilis*. These results indicated a bactericidal nature of LTP10 metabolites against gram-positive bacteia and a bacteriostatic nature against gram negative baceria. Similarly, Khim et al. reported MIC values of more than 2 mg/mL for ethyl acetate and chloroform extracts of *Alcaligenes faecalis* against *E. psidii* [66]. Inturri et al. determined MIC of cell free supernatants of *Bifidobacterium longum* and *Lactobacillus rhamnosus* against ATCC strains, including *E. coli*, *S. aureus*, and *P. aeruginosa* by the broth dilution method [69]. According to Shalayel et al., the MIC of ethyl acetate extract of *Mentha piperita* against all Gram-negative pathogens was as 5–40 mg/mL, with the lowest MIC (1.25 mg/mL) value against *Streptococcus pyogenes* [70]. When compared with standard antibiotic ampicillin, antimicrobial activity of the extract was lower, as evident by its higher MIC values (mg/mL vs µg/mL). The difference in potency is because of the complex nature of extract with varying concentrations of metabolites, while ampicillin is purely antibiotic. However, the extract exhibited activity against all tested organisms, demonstrating its broad spectrum potential.

The term “synergistic effect” denotes that a chemical combination's activity is better than the sum of its constituent compounds' individual effects. Antibiotics and secondary metabolites work together to combat many MDR pathogens [71]. For this reason, the combined antibacterial activity of ethyl acetate extract of LTP10 with few known antibiotics was determined by the agar disc diffusion method. It was found that ZOI of the extract increased when combined with penicillin, imipenem, and cefepime compared to extract alone against *S. aureus* and *E. coli*. The difference in ZOI of extract of LTP10 alone and in combination was statistically significant (P < 0.05). Keawchai et al. described the combined effect of a secondary metabolite obtained from ethyl acetate–hexane extract of *M. fragrans* seeds with ampicillin against *E. coli*, which greatly enhances the potential of ampicillin against *E. coli* [72]. Hussain et al. noted the synergistic antimicrobial effect of dinactin, a potent microbial metabolite, with drugs of tuberculosis against *Mycobacterium tuberculosis* [26]. The increase in ZOI indicated a potential interaction between extract and antiobiotics; however, as ZOI measurements indicate only qualitative assesment, further quantitative assays (e.g., MIC-based methods such as checkerboard assays) are required to confirm synergy.

Gas Chromatography-Mass spectrometry analysis were carried out to determine the antimicrobial secondary metabolites produced by *Alcaligenes pakistanensis* LTP10. Gas chromatogram of ethyl acetate extract showed various peaks, from which 22 compounds were tentatively identified based on NIST library matching. Moreover, the major metabolites in ethyl acetate extract have known bioactivities. For example, 2,4-Di-tert-butylphenol [53],[73], Dodecanoic acid, 3-hydroxy- [52], 7,9-Di-tert-butyl-1-oxaspiro (4,5)deca-6,9-diene-2,8-dione [55], Pyrimidine-2,4(1H,3H)-dione [50], Pentadecanoic acid,14-methyl-,methyl ester [51], 1-Nonadecanol [56], and Pyrrolo[1,2-a]pyrazine-1,4-dione, hexahydro-3-(phenylmethyl) [60] have reported good antimicrobial activities. Pyrrolo[1,2-a]pyrazine-1,4-dione,hexahydro-3-(2-methylpropyl) [74], and Pentadecanoic acid [55] have reported antioxidant activities. A study by Khim et al. reported a total of nine metabolites by GC-MS analysis of diethyl ether extract of *Alcaligenes faecalis* [66]. The metabolites were (1) di[1-(3,4- methylenedioxyphenyl)-2-propyl]amine, (2) clindamycin, (3) N-formylmaleamic acid, (4) malonic acid, (5) sabinene hydrate, (6) methyl salicylate, (7) methylethylketon, (8) gamma-Crotonolactone, and (9) valproic acid, and some metabolites detected in this extract were known to have antibacterial activities. Abd Sharad et al. also reported 2,4-Di-tert-butylphenol among 20 compounds in chloroform extract of *Alcaligenes feacalis* by GC-MS analysis [49]. The results of GC-MS chromatography in this study revealed that most tentatively identified metabolites from different extracts of *Alcaligenes pakistanensis* have antimicrobial effects against different types of microorganisms, while few have antioxidant activities reported. Thus, these results confirmed the results of antimicrobial assays performed in the study. It should be noted that GCMS based analysis, in the absence of authentic standards, provides putative identification of compounds based on library matching, with inherent limitations in structural elucidation.

FTIR was created as a technique for the simultaneous and quantitative measurement of organic components, including chemical bonds and organic content, such as proteins, carbohydrates, and lipids [75]. In this study, ethyl acetate extract of *A. pakistanensis* LTP10 was subjected to FTIR analysis, and the peaks at 3275, 2924, 1631, 1540, 1444, 1380, 1235, and 1071 cm^−1^ in the FTIR spectrum confirmed the presence of O-H, C-H, C=C, C-O, N-O, S=O, C-N, and C-O functional groups, respectively. These functional groups indicated the presesnce of organic compounds belonging to alcohols, aliphatic hydrocarbons, and nitrogen. These absorptions correlated well with the GCMS findings, which identified several oxygenated and hydrocarbon-based compounds, including long-chain fatty acid methyl esters (e.g., pentadecanoic acid, 14-methyl–methyl ester #8; heneicosanoic acid methyl ester #14), fatty alcohols (1-nonadecanol #17), hydroxy acids such as Dodecanoic acid, 3-hydroxy- (#10), and cyclic oxygenated dione structures such as 7,9-di-tert-butyl-1-oxaspiro(4,5)deca-6,9-diene-2,8-dione (#13). However, it should be noted that FTIR analysis identified the collective functional group profile of the crude extract, and the observed bands may also have arisen from minor or co-eluting metabolites, which were not confidently identified by GCMS. These functional groups supported the antimicrobial activity of extract, as several GCMS identified compounds have reported antibacterial and antifungal activities. *Alcaligenes faecalis'* crude ethyl acetate extract was FTIR-analyzed to reveal the presence of functional groups O-H, C-H, C-O, C=O, C=C, and N-O [76]. Similarly, FTIR analysis was used for methanolic extract of *Cnestis ferruginea*, and different functional groups were found in the FTIR spectrum of extract [77]. Pharmawati and Wrasiati performed the FTIR analysis of crude extract of *Enhalus acoroides* and found functional groups such as hydroxyl groups, secondary amines, alkanes, fatty acids, and phenols [78].

Furthermore, the molecular docking technique has been proved to be very effective in improving the efficiency of drug design and reducing research cost [37]. Compound selection was not confined to the most abundant metabolites in the extract but was based on multi-step in silico prioritization criteria, including drug likeness, safety profiling, predicted activity, and docking performance. Based on this criteria, two compounds, 7,9-Di-tert-butyl-1-oxaspiro(4,5)deca-6,9-diene-2,8-dione (Cpd 13) and Dodecanoic acid, 3-hydroxy- (Cpd 10), in ethyl acetate extract of LTP10 were selected based on their best results in preliminary screening by passing drug likeness filters and safety filters, and predicted pIC50 (4.54 to 5.13) and binding affinities (-5.5 to -7.8 kcal/mol) against target enzymes and reported antimicrobial activities. This systemic selection approach increased the probability of selecting true lead compounds with favorable drug likeness properties, acceptable safety profiles, and predictable biological activity to detailed docking and pharmacokinetic study. RMSD obtained after redocking of co-crystalized ligands trimethoprim against 7NAE (0.694 Å) and chemical fragment against 5Z9P (1.113 Å) validated the docking protocol followed for docking of sample compounds, as it was well below the acceptable threshold value of 2.0 Å [79]. Binding affinity of Cpd 13 and co-crystallized trimethoprim was -7.8 kcal/mol, defending strong antimicrobial activity of Cpd 13. Moreover, Cpd 13 and experimental inhibitor trimethoprim showed similar binding residues ILE50 and PHE31 with target 7NAE, further supporting the inhibitory effect of Cpd 13. Similarly, BA of Cpd 13 against 5Z9P was also comparable to experimental ligand chemical fragment. Additionally, R and S enantiomers of Cpd 10 showed good inhibitory activity against 7NAE and 5Z9P as evident from the presence of multiple hydrogen bonds and alkyl bonds at the binding sites. The bond length of most interactions was calculated as less than 3.5 Å, indicating a strong and stable binding affinity between the ligand and the active site residues [80]. The molecular docking and their detailed interaction studies of these compounds revealed their strong interference with the target bacterial enzymes, indicating their potential to inhibit microbial multiplication. However, experimental validation using pure compounds is required to confirm these findings.

The ADMET prediction of Cpd 13 and Cpd 10 by ADMETlab 3.0 and pkCSM revealed their important characteristics compared to the reference antibiotic trimethoprim. pkCSM predicted that human intestinal absorption of both compounds was higher than trimethoprim. None of the metabolites were predicted to be P-gp substrate or inhibitor, except trimethoprim as substrate, indicating lower risk of efflux-related reduction in intracellular concentration. BBB permeability of the compounds was less than that of trimethoprim, indicating its reduced CNS related side effects. Toxicity predictions showed no mutagenicity, hERG inhibition, skin sensitization, or significant hepatotoxicity for the metabolites, while trimethoprim showed comparatively higher hepatotoxicity risk. Moreover, the predicted ADMET properties of the two compounds are advantageous over trimethoprim, supporting their potential for further development as antimicrobial candidates, although experimental validation is required to confirm these computational analysis results.

Moreover, compounds Cpd 13 and Cpd 10 have qualified drug likeness rules like Lipinski, Veber, and Ghose filters, as predicted by SwissADME and ADMETlab 3.0, suggesting that these compounds have similar physicochemical properties to trimethoprim for oral drug development. Additionally, the synthetic bioavailability score of the compounds indicated their easy synthesis. Overall, the drug likeness assessment supports further development for both metabolites.

## 5. Conclusions

The results of antimicrobial activity, GCMS analysis, FTIR analysis, and in silico studies have confirmed the antimicrobial potential of crude extract of *Alcaligenes pakistanensis* LTP10. These findings provide new structural and functional insights into glacier-derived *Alcaligenes*, addressing a gap in research where such integrative metabolomic and computational analyses from Passu glacier isolates have not been reported. It was concluded that cold-adapted isolates from the underexplored Passu glacier could be considered a good source of antimicrobial compounds. However, it is advised that, in subsequent research, the extract be subjected to additional analytical processing to completely isolate and identify the antimicrobial metabolites, and more isolates from Passu glacier need to be explored for their antimicrobial potential. Additionally, the absence of whole genome sequencing and biosynthetic gene cluster analysis is a limitation, and to understand the biosynthetic potential of the isolate, researchers should incorporate genomic approaches.

## Use of AI tools declaration

The authors declare they have not used Artificial Intelligence (AI) tools in the creation of this article.
